# Humans frequently exposed to a range of non-human primate malaria parasite species through the bites of *Anopheles dirus* mosquitoes in South-central Vietnam

**DOI:** 10.1186/s13071-015-0995-y

**Published:** 2015-07-16

**Authors:** Yoshimasa Maeno, Nguyen Tuyen Quang, Richard Culleton, Satoru Kawai, Gaku Masuda, Shusuke Nakazawa, Ron P. Marchand

**Affiliations:** Department of Virology and Parasitology, Fujita Health University School of Medicine, 1-98 Kutsukake, Toyoake, Aichi 470-1192 Japan; Khanh Phu Malaria Research Unit, Medical Committee Netherlands-Viet Nam, Nha Trang, Khanh Hoa province Viet Nam; Malaria Unit, Institute of Tropical Medicine, Nagasaki University, Nagasaki, Nagasaki Japan; Laboratory of Tropical Medicine and Parasitology, Dokkyo Medical University, Mibu, Tochigi Japan; The Graduate School of Global Environmental Studies, Kyoto University, Kyoto, Kyoto Japan; Department of Protozoology, Institute of Tropical Medicine, Nagasaki University, Nagasaki, Nagasaki Japan

**Keywords:** Sporozoites, *Anopheles dirus*, *Plasmodium vivax*, *Plasmodium falciparum*, *Plasmodium knowlesi*, *Plasmodium cynomolgi*, *Plasmodium coatneyi*, *Plasmodium inui*

## Abstract

**Background:**

Recent studies have described natural human infections of the non-human primate parasites *Plasmodium knowlesi* and *Plasmodium cynomolgi*. In Southeast Asia, mosquitoes of the *Anopheles leucosphyrus* group bite both humans and monkeys in the forest and thus offer a possible route for *Plasmodium* species to bridge the species barrier. In this study we analysed the species composition of malarial sporozoites infecting the salivary glands of *Anopheles dirus* in order to determine their potential role as bridge vectors of *Plasmodium* parasites from monkeys to humans.

**Methods:**

Mosquitoes were collected in the forest and forest fringe area of Khanh Phu commune by human-baited landing collection. *Anopheles* species were determined on the basis of morphologic features. Sporozoite-infected salivary glands were applied to filter paper and dried in an ambient atmosphere, before storage in closed vials at 4–6 °C. Detection and identification of *Plasmodium* species in salivary glands were carried out by nested-PCR of the small subunit ribosomal RNA gene.

**Results:**

Six species of *Plasmodium* parasites were detected by PCR, of which *P. vivax* was the most common, followed by *P. knowlesi*, *P. inui*, *P. cynomolgi*, *P. coatneyi* and *P. falciparum*. Twenty-six of the 79 sporozoite infected mosquitoes showed multiple infections, most of which were a combination of *P. vivax* with one or more of the non-human primate *Plasmodium* species.

**Conclusions:**

These results suggest that humans overnighting in this forest are frequently inoculated with both human and non-human primate malaria parasites, leading to a situation conducive for the emergence of novel zoonotic malaria.

**Electronic supplementary material:**

The online version of this article (doi:10.1186/s13071-015-0995-y) contains supplementary material, which is available to authorized users.

## Background

Six malaria parasite species, *Plasmodium falciparum*, *Plasmodium vivax*, *Plasmodium malariae*, *Plasmodium ovale wallikeri, Plasmodium ovale curtisi* and *Plasmodium knowlesi* cause disease in humans. In Southeast Asia 13 species of *Plasmodium* parasites are found in non-human primates [1]. One of these, *P. knowlesi,* is now a well-known threat to human health in multiple countries in the region [2–6]. Recently, the first naturally acquired human infection of *Plasmodium cynomolgi* was described from the east coast of Peninsular Malaysia [7]. Little is known regarding the ability of other non-human primate *Plasmodium* parasites, besides *P. knowlesi* and *P. cynomolgi*, to infect humans, though *Plasmodium inui*, *Plasmodium eylesi*, *Plasmodium schwetzi* and others have been recorded as possessing the ability to infect humans as a result of experimental infection [8–16].

Malaria parasites have the ability to switch hosts [17], indeed, it is now thought that of the six species that commonly infect humans, at least three, *P. falciparum*, *P. vivax* and *P. knowlesi* were originally parasites of non-human primates that jumped the species barrier to man.

Zoonotic malaria infections can only occur when the vectors of non-human malaria parasites come into contact with people. Members of the *Anopheles dirus* complex are known to be important vectors of human *Plasmodium* parasites in the forests of Southeast Asia, and *An. dirus* (Species A) has previously been shown to vector *P. knowlesi* in Vietnam [6, 18, 19]. In order to assess the risk of potential zoonotic infections with non-human primate parasites, we assayed the malaria parasite species composition of sporozoites residing in the salivary glands of forest-caught *An. dirus* mosquitoes. Here we report the occurrence of *P. inui*, *P. cynomolgi* and *Plasmodium coatneyi* in the salivary glands of human-biting *An. dirus* mosquitoes in Vietnam.

## Methods

### Parasites

Positive control genomic DNA (gDNA) for *P. falciparum* was obtained from 3D7-9A *in vitro* cultured parasites, whilst *P. vivax* and *P. malariae* gDNA was extracted from Giemsa’s solution stained blood films [20]. *P. knowlesi* H strain (ATCC No. 30158) gDNA, obtained from an experimentally infected Japanese macaque (*Macaca fuscata*) [21] was used as a positive control. Twenty-five μL aliquots of clone 3D7-9A or *P. knowlesi* H strain infected blood was placed in a micro-reaction tube and kept at −80 °C until PCR analysis. The gDNA from *P. cynomolgi* B strain (ATCC No.30129), *P. inui* Taiwan II strain (ATCC No.30200) and *P. coatneyi* CDC strain was used as a positive control, respectively. These parasites were maintained in Japanese macaques in Dokkyo Medical University and cryopreserved in liquid nitrogen. Throughout the course of the animal experiments, investigators adhered to the Guidelines for the Use of Experimental Animals authorized by the Japanese Association for Laboratory Animal Science. The protocol was approved by the Committee on the Ethics of Animal Experiments of Dokkyo Medical University (Permit Number: 0656).

### Mosquito collection and dissection of salivary glands

Mosquitoes were collected by human-baited landing catches in the forest and forest fringe areas near Nga Hai village in the southern part of Khanh Phu commune, Khanh Vinh district, Khanh Hoa province, Vietnam. Khanh Phu is a commune with around 3,000 inhabitants, mainly of the Raglai ethnic minority, who live between the forested foothills on the east side of the Truong Son mountain range in south central Vietnam, an area where malaria was previously hyper- to holo-endemic.

Mosquitoes were collected in the forest south of Khanh Phu (12°11′N; 108°55′E) at a place where local people regularly enter the forest, from February 2010 to April 2013. Mosquito collectors were adult men of the Raglai ethnic group. They were regularly screened for malaria and promptly treated with artemisinin combination therapy if infected. The collectors worked in teams of two over the whole night, one person collecting from 18:00 to 24:00 h and the other from 0:01 to 6:00 h. The monthly average collection effort ranged between 18 and 48 person-nights per month, a total of 1,617 person-nights over the 36 month period (Table 1).

**Table 1:** Results of the collection, dissections and PCR processing of *Anopheles dirus* mosquitoes caught by human landing catch in “the forest” and “forest fringe”

*An. dirus* caught in the study area							
Year	Period	No. caught	No. nights	Biting density*	No. dissected	No. sporozoites	%
2010	Feb to Dec	2614	507	5.2	2613	48	1.8
2011	Jan to Dec	1584	549	2.9	1583	21	1.3
2012	Jan to Nov	1757	525	3.4	1757	16	0.9
2013	Mar to Apr	116	36	3.2	109	1	0.9
Feb/2010 to Apr/2013		6071	1617	3.8	6062	86	1.4
*An. dirus* caught in the forest							
2010	Feb to Dec	1668	303	5.5	1667	33	2
2011	Jan to Dec	1093	329	3.3	1092	15	1.4
2012	Jan to Nov	920	283	3.3	920	9	1.0
2013	Mar to Apr	27	10	2.7	26	1	3.8
Feb/2010 to Apr/2013		3708	925	4.0	3705	58	1.6
*An. dirus* caught in forest fringe							
2010	Feb to Dec	946	204	4.6	946	15	1.6
2011	Jan to Dec	491	220	2.2	491	6	1.2
2012	Jan to Nov	837	242	3.5	837	7	0.8
2013	Mar to Apr	89	26	3.4	83	0	0.0
Feb/2010 to Apr/2013		2363	692	3.4	2357	28	1.2

*Biting density, average human-biting density (No. of caught / No. of caught person-night)

*Anopheles* species were determined on the basis of morphology [22]. All *An. dirus* group mosquitoes were assumed to be *An. dirus* species A on the basis of previous accurate identifications and the known distribution of this species [23, 24]. Furthermore, a subset of 20 mosquitoes were analysed by PCR and sequencing, and all were shown to be of *An. dirus* species A (Additional file 1: Figure S1). Female anopheline mosquitoes were dissected for salivary glands, midguts and ovaries and these were examined by microscopy for sporozoites, oocysts and parity, respectively. Sporozoite-infected salivary glands were applied to filter paper and dried in an ambient atmosphere, before storage in closed vials at 4–6 °C.

### gDNA extraction and PCR detection

Extraction of gDNA from sporozoite-infected salivary glands on filter paper and subsequent PCR analysis was carried out as previously described [6, 18]. Briefly, gDNA was extracted using QIAamp DNA Micro Kit (Qiagen, Tokyo, Japan). *Plasmodium* species-specific nested-PCR assays to identify human and simian *Plasmodium* species were performed as previously described [2, 25, 26]. The genus-specific primers, rPLU-1/rPLU-5, were used in the primary amplification (nest 1) and performed as described by Singh *et al.* (1999) [25]. Detection of species-specific 18S rRNA genes (nest 2) was performed as previously described [2, 25, 26]. For the nest 2, 2 μL of 50× nest 1 amplification product was used as the template in the reaction mixtures (25 μL). For authenticating *P. knowlesi* infection, detection of the circumsporozoite protein (CSP) gene of *P. knowlesi* from samples was carried out as previously described by Vythilingam *et al.* (2008) [27]. A 2720 Thermal cycler (ABI, Foster city, CA, USA) was used for all PCRs. PCR products were separated by electrophoresis on 1.5 % agarose gels and stained with ethidium bromide. DNA bands were analysed with Lane & Spot Analyzer software (Atto, Tokyo, Japan). No amplification was observed with mosquito gDNA controls. Primer sequences for *18SrRNA* of human and non-human primate *Plasmodium* species-specific primers [2, 25, 26], and the CSP gene of *P. knowlesi* [27] were as previously described.

### Sequencing and analysis of DNA for *18SrRNA* of monkey *Plasmodium* species

For nucleotide sequencing, the specific products resulting from PCR amplification of the *18S rRNA* of non-human primate *Plasmodium* species were cleaned using the Wizard SV Gel and PCR Clean-up System (Promega, Tokyo, Japan) according to the manufacturer’s instructions, and were then sequenced with the BigDye Terminator v3.1 Cycle Sequencing Premix Kit (ABI). The reaction products for sequencing were separated with an ABI/Hitachi 3130x1 Genetic Analyzer (ABI) and the resulting nucleotide sequences were compiled using Genetyx (Genetyx Corporation, Tokyo, Japan). A phylogenetic tree constructed using these sequences is shown in Additional file 2: Figure S2.

### Statistical analysis

Statistical evaluation was performed using with the *X*^2^-test or the Mann–Whitney test (two-tailed). All analyses were performed using SPSS software (SPSS Japan, Tokyo, Japan), values of *P* < 0.05 was considered significant.

## Results

### *Plasmodium* parasite infections in *An. dirus* mosquitoes

During this period, as well as over many years before in this forest, *An. dirus* was the only anopheline species found with sporozoites. *An. dirus* mosquitoes were found during every month of sampling with an average human-biting density of 3.8 bites/person-night (range 2.9–5.2) in this study period. This human-biting density did not show significant differences between mosquito collection sites (Table 1).

A total of 6,071 female *An. dirus* were captured of which 6,062 were dissected for the detection of sporozoites in salivary glands by microscopic examination and 86 (1.4 %) were infected with *Plasmodium* sporozoites. Of the mosquitoes infected with sporozoites, 58 out of 3,705 (1.6 %) were found in the forest and 28 out of 2,357 (1.2 %) in the forest fringe. Both the biting densities as well as the infection rates were not significantly different between the sites of collection (Table 1).

### Identification of *Plasmodium* species in mosquito salivary glands

Of the 86 sporozoite-positive mosquitoes, 83 underwent nested PCR analysis for identification of species of *Plasmodium* sporozoites, of which 79 were successfully assayed. All of the PCR positive sporozoite samples were identified to the *Plasmodium* species level.

It has been shown previously that the *18sRNA* primer set specific for *P. knowlesi* can occasionally cross-react with *P. vivax* genomic DNA [28]. Therefore, identification of *P. knowlesi* was determined by three different PCR assays, and confirmed by sequencing of the products. Seventeen out of 79 PCR-positive samples were confirmed as *P. knowlesi* through sequencing of the 18S rRNA gene.

### Prevalence of *Plasmodium* species infections

Using a nested PCR assay, we detected six species of *Plasmodium* parasites, with *P. vivax* being most common (prevalence of 43 %), followed by *P. knowlesi* (15 %), *P. inui* (15 %), *P. cynomolgi* (10 %), *P. coatneyi* (9 %) and *P. falciparum* (8 %). *Plasmodium malariae* and *P. ovale* were not detected (Table 2).

**Table 2:** Summary of *Plasmodium* spp. infections in *Anopheles dirus*

Infection	*Plasmodium* spp.	No. of mosquitoes infected
Single (53)	Pf	4
	Pv	27
	Pk	4
	Pct	7
	Pcy	6
	Pin	5
Double (19)	Pf, Pv	3
	Pf, Pin	1
	Pv, Pk	7
	Pv, Pcy	3
	Pv, Pin	3
	Pk, Pin	2
Triple (6)	Pf, Pv, Pin	1
	Pv, Pk, Pcy	1
	Pv, Pk, Pin	2
	Pv, Pct, Pin	1
	Pk, Pct, Pin	1
Quadruple	Pv, Pct, Pcy, Pin	1
No. of PCR-positive		79
No. of PCR-negative		4
Total no. of examined sporozoite-positive		83

Pf = *P. falciparum*, Pv = *P. vivax*, Pk = *P. knowlesi*, Pct = *P. coatneyi*, Pcy = *P. cynomolgi*, Pin = *P. inui*

**Fig. 1:**
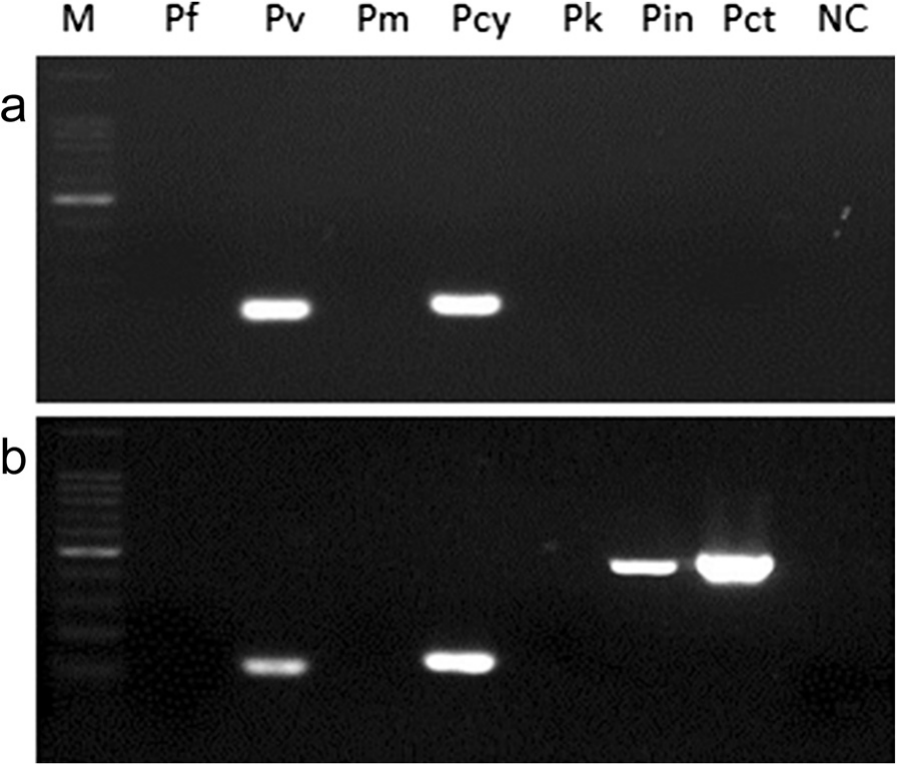
Detection of *Plasmodium* species-specific DNA from dried mosquito salivary gland of sample #118 (**a**) and 126 (**b**). The nested PCR assay was carried out for the detection of rRNA gene for *P. falciparum*, *P. vivax*, *P. knowlesi*, *P. coatneyi*, *P. cynomolgi*, and *P. inui*. M, 100 bp ladder size marker; Pf, *P. falciparum*; Pv, *P. vivax*; Pm, *P. malariae*; Pcy, *P. cynomolgi*; Pk, *P. knowlesi*; Pin, *P. inui*; Pct, *P. coatneyi*; NC, Negative control (*Plasmodium* DNA as the template was replaced with water)

Fifty-three out of 79 mosquitoes were positive for single *Plasmodium* species. Among them, *P. vivax* was dominant (prevalence of 51 %), followed by *P. coatneyi* (13 %), *P. cynomolgi* (11 %), *P. inui* (9 %), *P. falciparum* (8 %) and *P. knowlesi* (8 %) (Table 2). Co-infections of *Plasmodium* species were common, with 26 of the 79 (33 %) mosquitoes being infected by two or more species of *Plasmodium* each, most of the cases being mixed infection with *P. vivax* and another species (Fig. 1, Table 2). As shown in Table 2, *P. vivax* co-infections with the non-human primate *Plasmodium* species, *P. knowlesi* (33 %), *P. inui* (27 %), *P. cynomolgi* (17 %) and *P. coatneyi* (7 %), were observed. For *P. falciparum*, co-infection with non-human primate *Plasmodium* species was observed only with *P. inui* (7 %). In contrast, co-infection among human *Plasmodium* species was observed in only two cases, with *P. vivax* and *P. falciparum* (10 %) (Table 2).

### Comparison of *Plasmodium* species infections by collection site and biting time

**Fig. 2:**
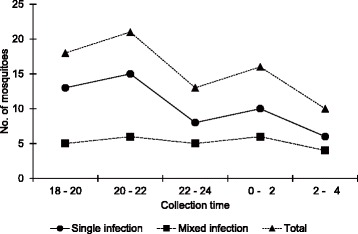
The biting rhythm of *An. dirus* infected with *Plasmodium* parasite in the forest. Single infection, single *Plasmodium* species infection; mixed infection, mixed *Plasmodium* species infections; Total, single infection + mixed infection

Although the data show a tendency towards more mosquitoes being infected with *P. cynomolgi* or *P. inui* in the forest compared with the forest fringe (Table 3), none of the differences between collection sites were significant. *Anopheles dirus* started to bite humans from right after sunset with a peak from 20:00 to 22:00 h and with a second peak from 0:00 to 2:00 h (Fig. 2). There were no significant differences in biting times between the forest fringe and the forest collection sites, or in the *Plasmodium* species composition at different times of the night.

**Table 3:** Comparison of *Plasmodium* spp. infections in *Anopheles dirus* by the collection sites

Infection	*Plasmodium* spp.	No. of mosquitoes infected	
		Forest fringe	In the forest
Single	Pf	1	3
	Pv	10	17
	Pk	1	3
	Pct	4	3
	Pcy	0	6
	Pin	1	4
Double	Pf, Pv	1	2
	Pf, Pin	0	1
	Pv, Pk	3	4
	Pv, Pcy	0	3
	Pv, Pin	2	1
	Pk, Pin	0	2
Triple	Pf, Pv, Pin	1	0
	Pv, Pk, Pcy	0	1
	Pv, Pk, Pin	0	2
	Pv, Pct, Pin	1	0
	Pk, Pct, Pin	0	1
Quadruple	Pv, Pct, Pcy, Pin	1	0
No.of examined sporozoite-positive		26	53

Pf = *P. falciparum*, Pv = *P. vivax*, Pk = *P. knowlesi*, Pct = *P. coatneyi*, Pcy = *P. cynomolgi*, Pin = *P. inui*

## Discussion

We evaluated the transmission of *Plasmodium* parasites by *An. dirus* in a forested area in the Khanh Phu commune, Vietnam, where previous studies demonstrated their co-infection with *P. knowlesi*, *P. falciparum*, *P. vivax* and *P. malariae.* [6, 18]. Our results suggest that both humans and macaques are commonly bitten by *An. dirus* in the forests around the Khanh Phu commune. To obtain data to verify whether and which macaques are the reservoirs of non-human primate *Plasmodium* parasites we previously collected and analysed macaque faecal samples collected from the forest floor, and from captive macaques in a cage in the forest where *An. dirus* are commonly found. Caged macaques were infected with *P. cynomolgi, P. coatneyi*, *P. inui and P. knowlesi,* with the latter species detectable only in faecal samples [29]. The data presented here show that *An. dirus* in the Khanh Phu forest are infected with two species of human *Plasmodium* parasites, *P. falciparum* and *P. vivax*, and four species of non-human primate *Plasmodium* parasites, *P. knowlesi*, *P. inui*, *P. cynomolgi* and *P. coatneyi*. Approximately one-third of mosquitoes were infected by two or more species of *Plasmodium,* and these were often a combination of human and non-human primate parasites. The numerous co-infections and the lack of differences between collection sites and biting times in addition suggest that all these parasites are transmitted by just one population of *An. dirus*, which bite humans as readily as macaques.

Among the non-human primate *Plasmodium* parasite sporozoites identified in this study, *P. knowlesi* dominated, followed by *P. inui*, *P. cynomolgi* and *P. coatneyi*. Several studies in Southeast Asia have demonstrated similar prevalences of non-human primate *Plasmodium* species in macaques. In Singapore, *P. knowlesi* was the most common species followed by *P. cynomolgi* and *P. inui* [30]. In Malaysian Borneo, however, the dominant species was *P. inui* followed by *P. knowlesi*, *P. coatneyi* and *P. cynomolgi* [26].

The results of this study indicate that humans overnighting in Khanh Phu forest are frequently inoculated with a range of simian *Plasmodium* parasites, several of which have been shown to be capable of causing disease in humans [2–8, 11, 14, 15, 31]. The implications of this situation are potentially serious. As it is known that malaria parasites are capable of jumping species and causing zoonotic infections in certain circumstances, the major factor governing the chances of a new zoonotic species emerging is exposure. The greater the number of inoculations into humans of macaque parasites, and the greater the number and diversity of species being introduced, the higher the chance becomes of either a permissible human host being encountered, or the inoculation of a mutant parasite capable of causing a zoonotic infection. Situations such as the one identified here, in which humans are routinely and regularly exposed to inoculations of (currently) non-human primate sporozoites, are likely to be highly conducive to the emergence of novel zoonotic malaria infections, and should be prevented as much as possible and monitored closely.

## Conclusion

We report the detection of sporozoites of both human and non-human primate *Plasmodium* parasites from the same mosquito population. Six species of *Plasmodium* were detected by PCR, of which *P. vivax* was the most common, followed by *P. knowlesi*, *P. inui*, *P. cynomolgi*, *P. coatneyi* and *P. falciparum*. About one-third of sporozoite infected mosquitoes showed multiple infections, most of which were a combination of *P. vivax* with one or more of the non-human primate *Plasmodium* species. These results suggest that humans overnighting in this forest are frequently inoculated with these malaria parasites, leading to a situation conducive for the emergence of novel zoonotic malaria parasites.

## Additional files

Additional file 1: Figure S1. Phylogenetic tree showing how the mosquitoes considered in this work relate to other members of the *Anopheles dirus* complex. We amplified and sequences a bp fragments of ribosomal DNA internal transcribed spacer 2 gene from a subset of 20 mosquitoes identified as *Anopheles dirus* species A. As a result of BLAST analysis, sequence of KP samples showed 99 % homology to *An. dirus* A (U60410), and in contrast, only 97 % homology to *An. dirus* D. The evolutionary history was inferred using the Neighbor-Joining method. The percentage of replicate trees in which the associated taxa clustered together in the bootstrap test (1000 replicates) are shown next to the branches. The tree is drawn to scale, with branch lengths in the same units as those of the evolutionary distances used to infer the phylogenetic tree. The evolutionary distances were computed using the Kimura 2-parameter method and are in the units of the number of base substitutions per site. Evolutionary analyses were conducted in MEGA5. Additional file 2: Figure S2. Phylogenetic tree based on the sequence of the 18sSSUrRNA gene of malaria parasites isolated from mosquitoes. The evolutionary history was inferred using the Neighbor-Joining method. The percentage of replicate trees in which the associated taxa clustered together in the bootstrap test (1000 replicates) are shown next to the branches. The tree is drawn to scale, with branch lengths in the same units as those of the evolutionary distances used to infer the phylogenetic tree. The evolutionary distances were computed using the Kimura 2-parameter method and are in the units of the number of base substitutions per site. Evolutionary analyses were conducted in MEGA5. By the BLAST analysis, sequences of *P. inui* and *P. coatneyi* was asexual stage (A type) and that of *P. cynomolgi* and *P. knowlesi* was sexual stage (S type).
