# Supplementary figures and images for: Humans frequently exposed to a range of non-human primate malaria parasite species through the bites of Anopheles dirus mosquitoes in South-central Vietnam
Source: Parasit Vectors. 2015 Jul 16;8:376. doi: 10.1186/s13071-015-0995-y (PMC4504216; doi:10.1186/s13071-015-0995-y)

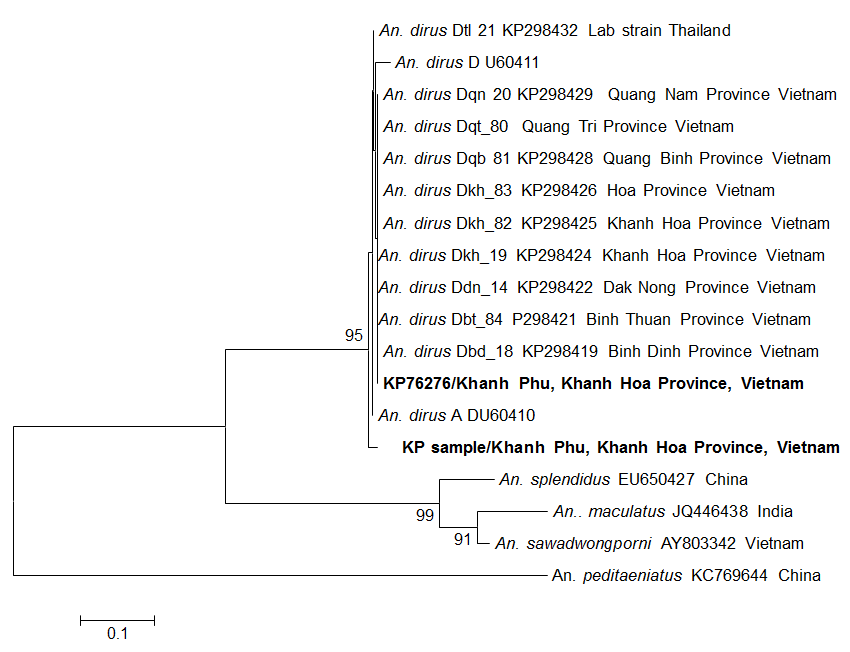

Supplement: Additional file 1: Figure S1. — Phylogenetic tree showing how the mosquitoes considered in this work relate to other members of the Anopheles dirus complex. We amplified and sequences a bp fragments of ribosomal DNA internal transcribed spacer 2 gene from a subset of 20 mosquitoes identified as Anopheles dirus species A. As a result of BLAST analysis, sequence of KP samples showed 99 % homology to An. dirus A (U60410), and in contrast, only 97 % homology to An. dirus D. The evolutionary history was inferred using the Neighbor-Joining method. The percentage of replicate trees in which the associated taxa clustered together in the bootstrap test (1000 replicates) are shown next to the branches. The tree is drawn to scale, with branch lengths in the same units as those of the evolutionary distances used to infer the phylogenetic tree. The evolutionary distances were computed using the Kimura 2-parameter method and are in the units of the number of base substitutions per site. Evolutionary analyses were conducted in MEGA5. [file 13071_2015_995_MOESM1_ESM.tif]

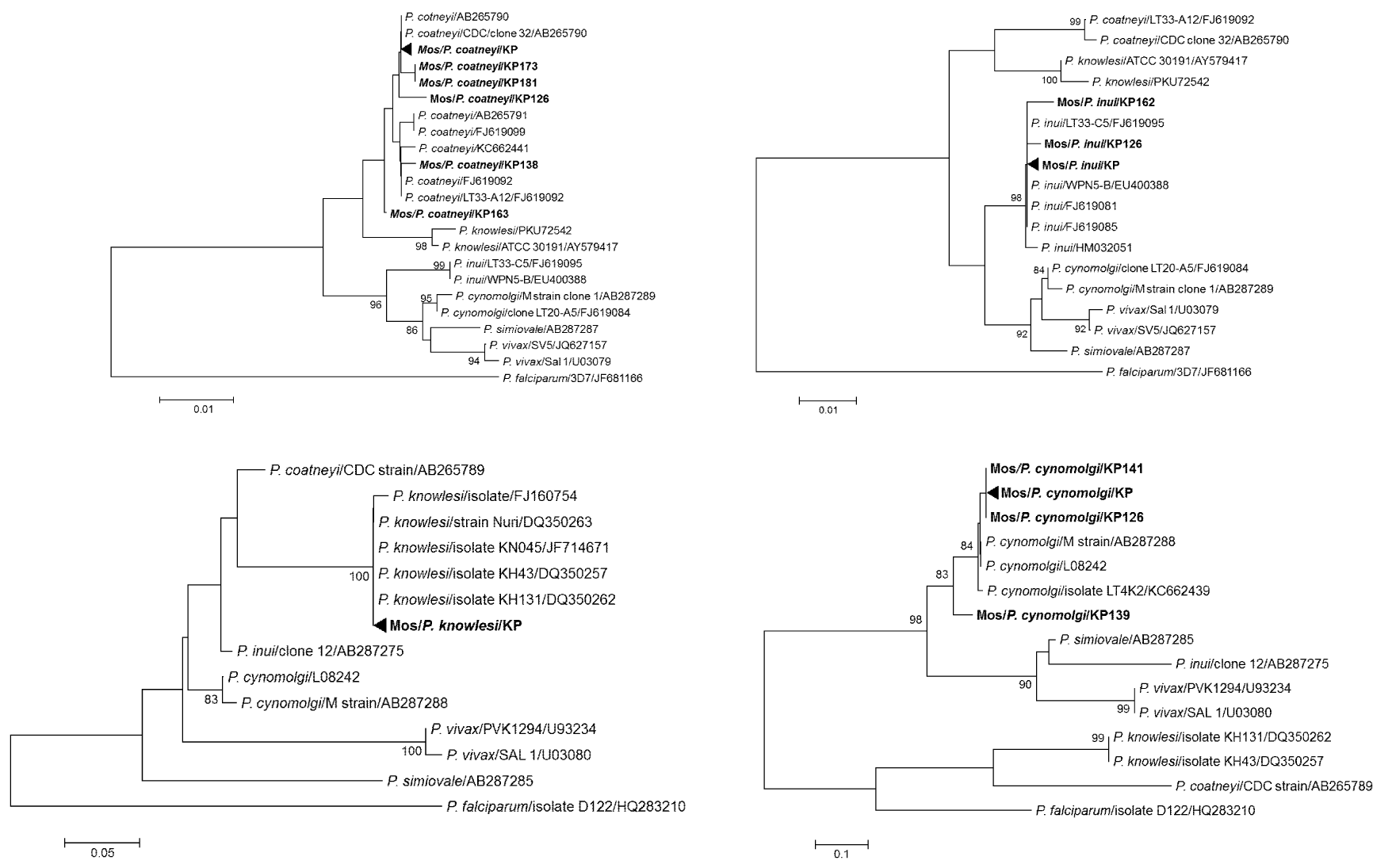

Supplement: Additional file 2: Figure S2. — Phylogenetic tree based on the sequence of the 18sSSUrRNA gene of malaria parasites isolated from mosquitoes. The evolutionary history was inferred using the Neighbor-Joining method. The percentage of replicate trees in which the associated taxa clustered together in the bootstrap test (1000 replicates) are shown next to the branches. The tree is drawn to scale, with branch lengths in the same units as those of the evolutionary distances used to infer the phylogenetic tree. The evolutionary distances were computed using the Kimura 2-parameter method and are in the units of the number of base substitutions per site. Evolutionary analyses were conducted in MEGA5. By the BLAST analysis, sequences of P. inui and P. coatneyi was asexual stage (A type) and that of P. cynomolgi and P. knowlesi was sexual stage (S type). [file 13071_2015_995_MOESM2_ESM.tif]
